# One-step technique for endoscopic ultrasound-guided hepaticogastrostomy using a partially covered metal stent for superficial bile duct access

**DOI:** 10.1055/a-2361-1412

**Published:** 2024-07-29

**Authors:** Takeshi Ogura, Yuki Uba, Nobuhiro Hattori, Kimi Bessho, Hiroki Nishikawa

**Affiliations:** 1 13010 2nd Department of Internal Medicine, Osaka Medical and Pharmaceutical University, Takatsuki, Japan; 2 13010 Endoscopy Center, Osaka Medical and Pharmaceutical University, Takatsuki, Japan

Endoscopic ultrasound-guided hepaticogastrostomy (EUS-HGS) is clinically useful as an alternative biliary drainage technique for patients with failed endoscopic retrograde cholangiopancreatography (ERCP) 1 ; however, one of the adverse events is biliary peritonitis due to bile leakage during the EUS-HGS procedure. Although biliary peritonitis is usually treated conservatively, it can worsen a patient’s condition by delaying oral intake or causing fever postoperatively 2 .

Biliary peritonitis can occur as a complication of intraoperative bile leakage, which among the steps involved in EUS-HGS, may be caused by tract dilation. To prevent this adverse event and to obtain a tamponade effect, enough hepatic parenchyma, at least 2.5 cm, should be crossed when the bile duct is punctured 3 . Another strategy, stent deployment without tract dilation, which is called the ‘one-step technique,’ is also sometimes considered 4 ; however, owing to the use of a fully covered stent, the one-step technique may cause potential stent dislocation or branch bile duct obstruction. Recently, a novel partially covered self-expandable metal stent (PCSEMS) has become available (HANARO Benefit, M.I Tech., Seoul, South Korea). This stent has a 1-cm uncovered portion and its stent delivery system is only 5.9 Fr ( Fig. 1 ). Therefore, insertion of this stent using the one-step technique may be feasible. Additionally, owing to the presence of the uncovered portion, the risk of stent dislocation or bile duct branch obstruction might be reduced. We herein describe the one-step technique for EUS-HGS using a PCSEMS with a fine gauge stent delivery system in a case requiring superficial bile duct access.

**Fig. 1:**
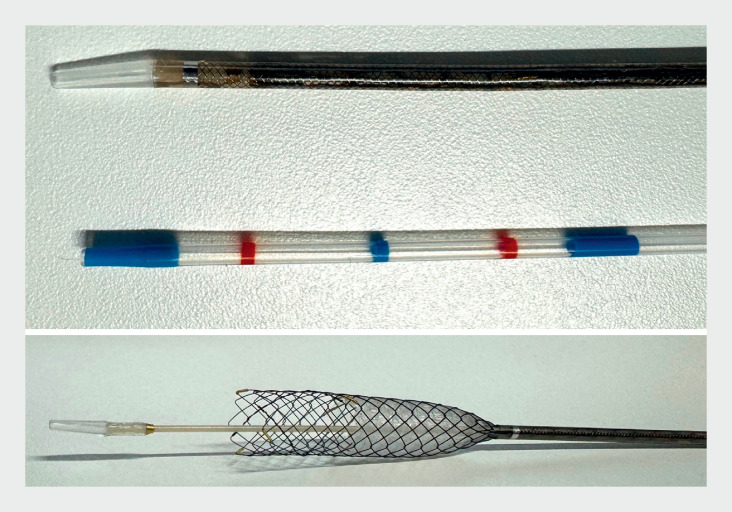
Photograph of the novel partially covered self-expandable metal stent, which has a 1-cm uncovered portion and a stent delivery system of only 5.9 Fr.

A 79-year-old man was admitted to our hospital with obstructive jaundice. He had undergone right hepatectomy because of a metastatic tumor caused by colon cancer. Owing to the surgically altered anatomy, EUS-HGS was attempted. As multiple liver metastases were observed, the only available puncture site was the superficial bile duct, access to which involved traversing a 16.0-mm length of the hepatic parenchyma ( Fig. 2 ). After a successful puncture had been performed, contrast medium was injected, with bile leakage observed ( Fig. 3 **a** ). Because additional tract dilation increases bile leakage, we decided to perform the one-step technique. The novel stent was successfully inserted into the biliary tract ( Fig. 3 **b** ) and successfully deployed using the intrascope channel release technique ( Fig. 3 **c** ; Video 1 ). Although the patient experienced mild abdominal pain postoperatively, no severe adverse events were observed.

**Fig. 2:**
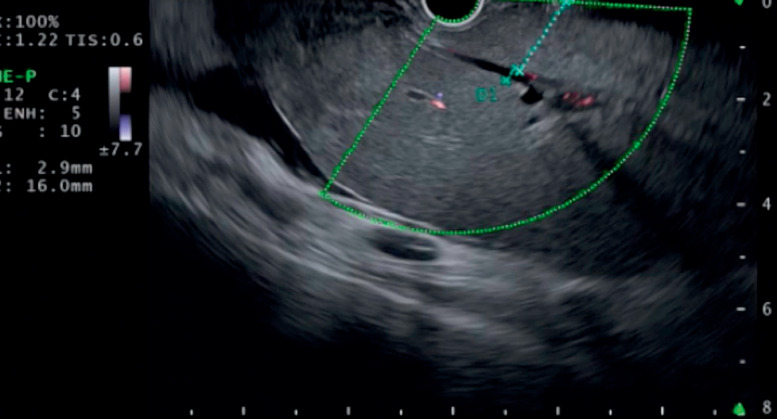
Endoscopic ultrasound image showing that the length of the hepatic parenchyma traversed to reach the puncture site was 16 mm.

**Fig. 3:**
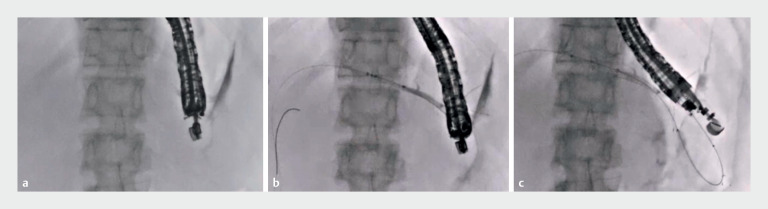
Fluoroscopic images showing: **a** leakage of bile observed after contrast medium injection; **b** the stent delivery system of the partially covered self-expandable metal stent after its successful insertion into the biliary tract; **c** deployment of the metal stent.

A novel partially covered self-expandable metal stent is deployed into a superficial bile duct without tract dilation for biliary obstruction.Video 1

In conclusion, the one-step technique using the novel PCSEMS might be useful in cases requiring superficial bile duct access.

Endoscopy_UCTN_Code_TTT_1AS_2AH

## Notes

Endoscopy E-Videos https://eref.thieme.de/e-videos
*E-Videos* is an open access online section of the journal *Endoscopy* , reporting on interesting cases and new techniques in gastroenterological endoscopy. All papers include a high-quality video and are published with a Creative Commons CC-BY license. Endoscopy E-Videos qualify for HINARI discounts and waivers and eligibility is automatically checked during the submission process. We grant 100% waivers to articles whose corresponding authors are based in Group A countries and 50% waivers to those who are based in Group B countries as classified by Research4Life (see: https://www.research4life.org/access/eligibility/ ). This section has its own submission website at https://mc.manuscriptcentral.com/e-videos .
